# Treatment of Greater Trochanteric Pain Syndrome With Ultrasound-Guided Bipolar Pulsed Radiofrequency of the Trochanteric Branches of the Femoral Nerve: A Case Series of Nine Patients

**DOI:** 10.7759/cureus.50422

**Published:** 2023-12-12

**Authors:** André Vieira, Mariana C Coroa, Noélia Carrillo-Alfonso, Francisco D Correia

**Affiliations:** 1 Physical Medicine and Rehabilitation, Hospital Central do Funchal, Funchal, PRT; 2 Anesthesiology, Centro Hospitalar de Vila Nova de Gaia e Espinho, Vila Nova de Gaia, PRT; 3 Anesthesiology, Centro Hospitalar Universitário do Algarve, Faro, PRT; 4 Pain Medicine, Hospital Central do Funchal, Funchal, PRT

**Keywords:** ultrasound-guided, ultrasound-guided bipolar pulsed radiofrequency, pain management, musculoskeletal pain, bipolar pulsed radiofrequency, greater trochanteric pain syndrome

## Abstract

Background: Greater trochanteric pain syndrome (GTPS) is a prevalent cause of lateral hip pain that often leads to significant functional limitations. Conservative treatment options include physical therapy, pharmacological treatment, and more invasive techniques such as corticosteroid injections. However, despite the high success rates reported with corticosteroid injections, a significant number of patients have their symptoms persist or recur.

Objectives: In this case series, we present the outcomes of nine patients with GTPS who underwent ultrasound-guided bipolar pulsed radiofrequency targeting the trochanteric branches of the femoral nerve. We aim to discuss the effectiveness and safety of this approach.

Material and methods: Eligible patients referred to our centre underwent ultrasound-guided bipolar pulsed radiofrequency aimed at the trochanteric branches of the femoral nerve. The procedure consisted of one cycle at 42°C for six minutes, followed by the injection of ropivacaine (0.2%, 3 mL) and dexamethasone (12 mg). The Brief Pain Inventory - Short Form (BPI-sf) and Lequesne Algofunctional Index (LAI) were used before the procedure and at the third and sixth months post-procedure. We monitored immediate and late complications, as well as adverse effects.

Results and discussion: Our results indicate a favourable outcome for most patients, with an average pain reduction of 76.51% according to their report of the BPI-sf. Additionally, eight out of nine patients experienced at least 50% relief. These findings align with a previous case series, which reported a similar average pain reduction. Before the procedure, most patients were classified as “extremely severe” in the LAI, with an average score of 18.17. Although there was only a slight reduction of 16.84% at the six-month follow-up, this suggests a potential improvement in their functional status. We did not observe any immediate complications or adverse effects after the procedure, nor were any reported at the subsequent follow-ups, which is consistent with existing literature.

Conclusions: Our study suggests that ultrasound-guided bipolar pulsed radiofrequency treatment is a promising minimally invasive technique for GPTS, especially for patients who do not respond to conservative treatments. Although our case series provides some evidence of effectiveness and safety, further controlled studies on a larger scale are necessary, particularly to compare this intervention with the use of corticosteroid injections alone.

## Introduction

Greater trochanteric pain syndrome (GTPS) presents as peritrochanteric pain in the lateral hip, often associated with tenderness during palpation [1, 2]. Historically misdiagnosed as trochanteric bursitis [3], it’s now recognised to encompass various conditions, including tendinopathy, tears of the gluteal tendons, trochanteric bursitis, and external coxa saltans [2,4,5].

This prevalent condition affects 1.8 to 5.6 patients per 1,000 per year [6, 7], with a prevalence of 10% to 25% among adults aged 40 to 60 years [3, 8].

It predominantly affects women (with a four-to-one female-to-male ratio), middle-aged adults, specific athletes, and individuals with hip osteoarthritis or low back pain [3, 8, 9].

Diagnosis relies on clinical findings of insidious lateral hip pain, occasionally intermittent and radiating laterally and posteriorly, exacerbated by sleeping on the affected side or bearing weight ipsilaterally [3, 5, 8]. Physical examination reveals tenderness around the greater trochanter, accompanied by positive provocative manoeuvres which are the Faber test, the resisted external derotation test, or resisted hip abduction [3, 5, 8, 10]. In some cases, imaging studies may be necessary to confirm the diagnosis and exclude other conditions.

Initial management of GTPS involves activity modification, rest, physical therapy, and non-steroidal anti-inflammatory drugs (NSAIDs) [11, 12]. For refractory cases, therapy with local corticosteroid injections is common [13, 14], although various other interventions like shockwave therapy, platelet-rich plasma injections, or percutaneous tendon fenestration have been described [9, 11, 15]. Surgical interventions are reserved for those exhausting non-surgical options, with recent literature detailing endoscopic techniques for such cases [9, 11, 16].

Despite the reported positive outcomes of conservative management in approximately 90% of cases [17], persistent or recurrent symptoms remain common, leading to incomplete relief or multiple courses of non-operative treatment [7]. A retrospective cohort study revealed that the majority of patients continued to experience pain in the first and fifth years of follow-up (76% and 63%, respectively) [18].

Radiofrequency treatment has shown success in treating chronic musculoskeletal pain [19], yet its potential for GTPS has not been explored. Notably, a 2012 study on cadavers identified the involvement of a small branch of the femoral nerve in the sensory innervation of the periosteum and bursae of the greater trochanter, suggesting a potential target for this intervention [20].

To address the persistent challenges in GTPS management, our study delves into the effectiveness and safety of ultrasound-guided bipolar pulsed radiofrequency targeting the trochanteric branches of the femoral nerve. We aim to evaluate the treatment's outcomes, presenting a case series involving nine patients diagnosed with GTPS, and comparing our findings with existing literature to contribute to a deeper understanding and potential advancement in the management of this syndrome.

## Materials and methods

We enrolled patients who met the criteria for greater trochanteric pain syndrome and were referred to our centre, a multidisciplinary pain centre located in the Hospital Central do Funchal, a tertiary healthcare facility in Funchal, Portugal. Diagnosis confirmation was based on typical findings of lateral hip pain accompanied by corresponding physical examination indications. These patients were referred to our unit within the public healthcare system after unsuccessful attempts with conservative treatments such as medication, physical therapy, and/or steroid injections.

We selected patients who had the diagnosis confirmed by our team, were refractory to first-line treatment options, and excluded patients who didn’t consent to the procedure or to be included in this study. The length of the study was six months.

Comprehensive details regarding the proposed procedure, encompassing potential risks and benefits, were communicated to all patients before the intervention. Informed consent was obtained from each participant, ensuring their complete understanding and voluntary participation.

**Figure 1:**
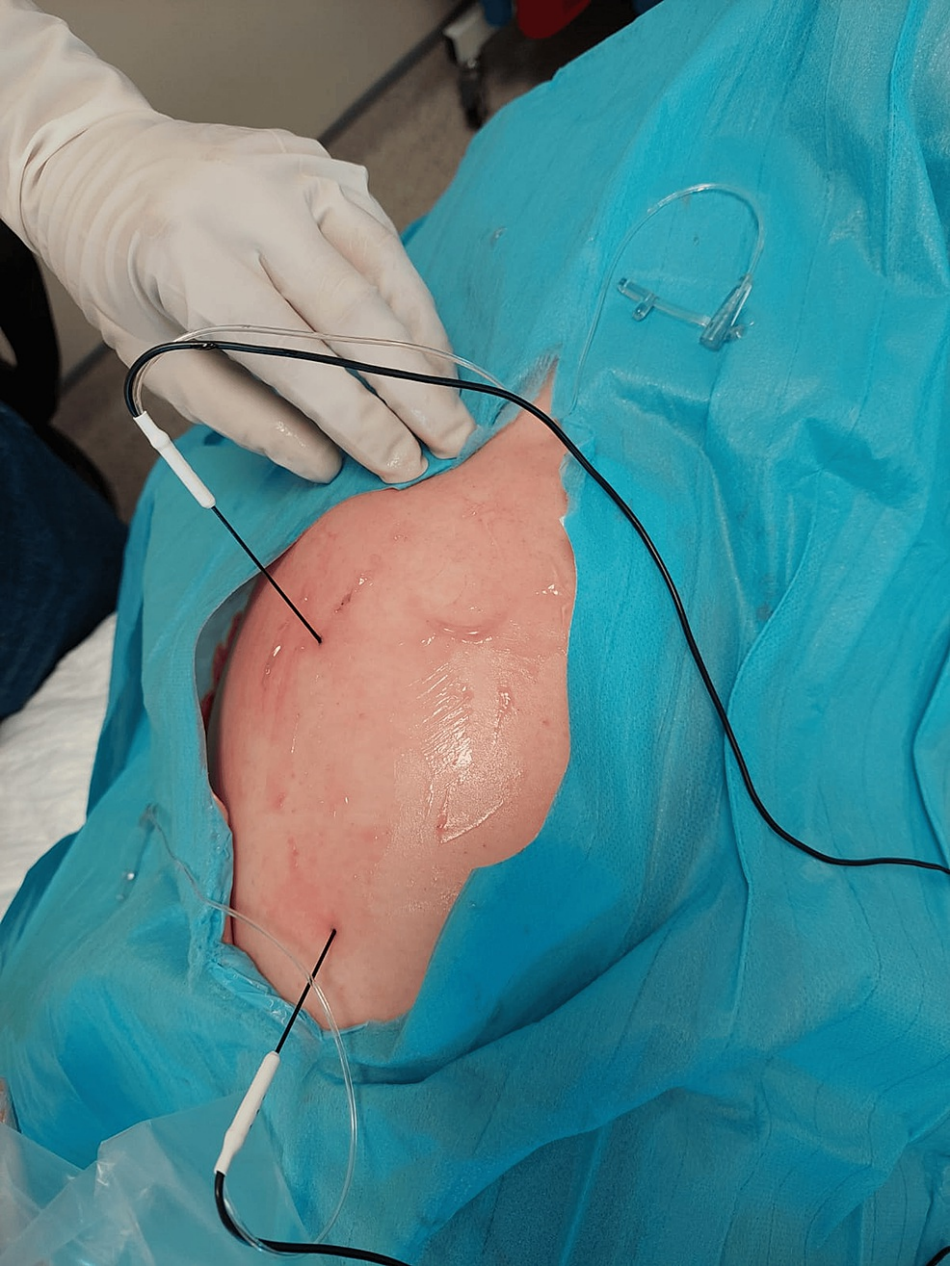
The image shows both the needle positions in the lateral hip of the patient undergoing treatment in case two.

**Figure 2:**
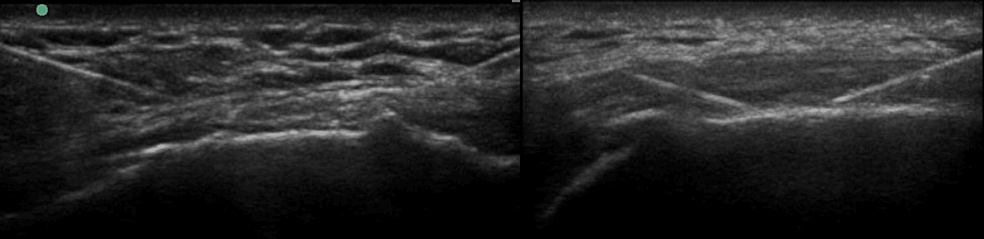
The image depicts the needle positioning with ultrasound guidance in cases three and five, respectively. The green dot indicates the cranial side (in both ultrasound pictures).

All procedures were performed with the patient positioned in the lateral decubitus, and a linear transducer was used to identify the greater trochanter. By moving the transducer posteriorly in a sagittal plane, we were able to visualise the posterior aspect of the greater trochanter. This is shown in Figures 1-2.

To ensure patient comfort, local anaesthesia with lidocaine (1%) was administered at the intended entry sites. Subsequently, two radiofrequency needles (straight 22-gauge, 10 cm, 10 mm active tip) were inserted less than 1 cm apart. Following needle placement, sensory testing showed positivity at ≤0.5 volts, while motor testing displayed negativity at 1.5 volts. Bipolar pulsed radiofrequency was administered at a single cycle of 42°C for six minutes, followed by the injection of ropivacaine (0.2%, 3 ml) and dexamethasone (12 mg).

To assess the intensity of pain and its impact on daily living, the Brief Pain Inventory -Short Form (BPI-sf) [21] was employed. Additionally, the Lequesne Algofunctional Index (LAI) [22] was employed to evaluate the impact on the patient’s daily life. Evaluations occurred pre-procedure and at three and six months post-procedure, with a follow-up telephone appointment scheduled one month after the procedure. Vigilant monitoring for immediate or delayed adverse effects was implemented.

## Results

Case one

A 79-year-old female presented with chronic lateral hip pain, severely limiting her walking ability. Initial assessment revealed a pain rating of 9/10 on the numerical rating scale (NRS) and LAI score of 19.5. Consequently, we opted for pulsed radiofrequency targeting the trochanteric branches of the femoral nerve. Upon the six-month follow-up, the patient reported a remarkable 66.66% reduction in current pain levels (3/10). However, she noted an average pain level of 5/10 over the preceding 24 hours, peaking at 8/10. Functionally, her LAI score was 16.5, indicating a slight improvement, particularly in walking distance impairment by one point. Overall, the patient perceived the intervention as beneficial.

Case two

A 43-year-old female, a mountain hiking guide, was referred to our clinic due to refractory bilateral greater trochanteric pain syndrome. At the time of referral, she was on sick leave due to her inability to walk long distances. Initially reporting a pre-procedural pain intensity of 8/10 on the NRS, which averaged at 6/10 over the preceding 24 hours, and an LAI score of 11, she primarily experienced more pronounced pain on the left side, while the right side exhibited minimal discomfort. Consequently, the procedure was performed on the left side. Remarkably, the patient remained pain-free throughout the six-month follow-up and resumed her professional activities without limitations. It is presumed that the resolution of pain on the right side was a result of an improved gait pattern.

Case three

A 63-year-old male driver initially sought pain relief for gonarthrosis while awaiting knee joint arthroplasty. Subsequently, he developed unilateral GTPS. Before the procedure, despite being prescribed oxycodone/naloxone (5 mg/2.5 mg) and Metamizole (575 mg), he maintained intense pain (7/10 on the NRS), reaching a maximum of 9/10 in the previous 24 hours. His LAI score was 18. Pulsed radiofrequency of the trochanteric branches of the femoral nerve was performed without complications. At the six-month follow-up, he reported a 71.43% reduction (2/10 on the NRS) in current pain levels.

Case four

A 79-year-old woman initially referred for incapacitating lower back pain (failed back surgery syndrome), with a medical history including obesity, gonarthrosis, and coxarthrosis, developed unilateral lateral hip pain, diagnosed as GTPS by our team. Pre-procedure pain was rated at 10/10 on the NRS, with average pain in the last 24 hours at 5/10 and an LAI score of 24. Pulsed radiofrequency of the trochanteric branches of the femoral nerve was performed without complications due to these factors. Six months later, the patient reported a 70% reduction (3/10 on the NRS) in pain levels and maintained an average pain score of 5/10, with a slight decrease in maximum pain in the last 24 hours to 7/10. Functionally, the LAI score was 20.

Case five

An 82-year-old male patient, previously treated for lower back pain and coxarthrosis at our medical centre, was diagnosed with GTPS and had undergone conservative treatment. However, GTPS became his recent primary complaint, with pain rated at 6/10 on the NRS and a maximum of 9/10 in the previous 24 hours, along with an LAI score of 12. He underwent pulsed radiofrequency of the trochanteric branches of the femoral nerve without complications. At the six-month follow-up, he reported being presently pain-free and experiencing an average and maximum pain level of 2/10 on the NRS in the last 24 hours. Functionally, his LAI score improved to 10.5. Due to the satisfactory pain reduction, his pain relief medication dosage was reduced, and a follow-up appointment was scheduled.

Case six

A 72-year-old female patient with a history of knee joint arthritis presented with bilateral GTPS. Pre-procedure, she rated her pain at 5/10 on the NRS, with an average pain over 24 hours at 7/10, a maximum of 9/10, and an LAI score of 20. Pulsed radiofrequency of the trochanteric branches of the femoral nerve was performed on her right hip without complications. At the six-month follow-up, there was a 42.86% reduction (3/10 on the NRS) in her pain level, with an average and maximum pain of 5/10 and 7/10, respectively. Her functional score on the LAI was 18. As the patient continued to experience similar complaints on her left side, a decision was made to repeat the procedure on the contralateral side as well.

Case seven

A 69-year-old woman, initially referred for coxarthrosis pain relief while awaiting hip joint arthroplasty, also reported ipsilateral GTPS and had been treated with oxycodone/naloxone (10 mg/5 mg). Before the procedure, her pain was rated at 6/10 on the NRS, averaging at 7/10 over 24 hours with a maximum of 9/10 and an LAI score of 21. Pulsed radiofrequency of the trochanteric branches of the femoral nerve was performed without adverse effects. At the six-month follow-up, she reported a 50% reduction in pain (4/10 on the NRS), with an average and maximum pain of 5/10 and 5/10, respectively. Functionally, her LAI score was 18. As the patient awaited a confirmed date for hip joint arthroplasty, the decision was made to schedule further treatment to alleviate her symptoms.

Case eight

A 79-year-old woman with a history of secondary multiple joint arthrosis caused by pseudogout was referred by the rheumatology department due to unilateral GTPS. Despite experiencing these symptoms for several years, she had not previously undergone the administration of a local corticosteroid injection. Therefore, as an initial intervention, we decided to perform that. There was no significant improvement after the corticoid injection, and she rated her pain as 6/10 on the NRS, with an average pain in the last 24 hours being 6/10, a maximum of 10/10, and a score of 20 in the LAI. Following that, she underwent pulsed radiofrequency of the trochanteric branches of the femoral nerve, which was conducted without any immediate or late adverse effects. At the six-month follow-up, she reported being pain-free at the time of the assessment. However, her average pain score over the previous 24 hours was 4/10 on the NRS, while the maximum pain remained consistently high at 10/10. Functionally, her LAI score had a slight reduction to 19. Despite the absence of current pain and a slight reduction in average pain, there was no improvement in the maximum pain level. Consequently, considering the persistently high pain levels and unaltered functional status, we opted to schedule shockwave therapy as the patient hadn’t achieved satisfactory results with the previous treatments.

Case nine

An 82-year-old male patient initially sought pain relief for gonarthrosis, later developing bilateral GTPS. Before the procedure, he rated his pain at 5/10 on the NRS, with an average pain of 5/10 over the last 24 hours and a maximum of 7/10. His LAI score was 18. Subsequently, pulsed radiofrequency targeting the trochanteric branches of the femoral nerve was performed on his right hip without any immediate or late adverse effects. At the six-month follow-up, the patient reported being pain-free, experiencing an average pain level of 1/10, with the maximum pain being 6/10 on the NRS over the previous 24 hours. Functionally, he scored 16.5 on the LAI. Despite this improvement, similar complaints persisted on his left side. Consequently, a decision was made to repeat the procedure on the opposite side as well.

In this case series, nine patients underwent pulsed radiofrequency targeting of the trochanteric branches of the femoral nerve for GTPS. Their average age was 70.44 years, comprising six females and three males. While most experienced unilateral symptoms, three reported bilateral pain. Given their average age and referral to a pain centre, concurrent active pain conditions were prevalent: 55% had lower back pain, 44.44% had gonarthrosis, and 66.66% had coxarthrosis.

Following the procedure, significant pain reduction was observed at the six-month follow-up compared to pre-procedural levels. On average, responses to the question "How much pain do you have right now?" decreased from 8.78 to 1.67 (76.51% reduction), with eight out of nine patients achieving more than 50% pain relief. However, other aspects assessed by the BPI-sf showed less substantial improvements, with an average reduction of 41.6% in "pain at its worst in the last 24 hours," 53.7% in "pain at its least in the last 24 hours," and 46.58% in "your pain on average."

**Figure 3:**
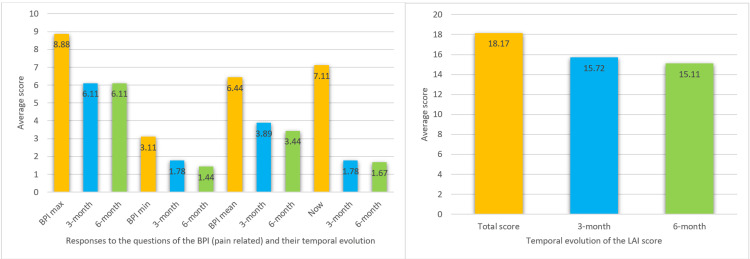
Average data of the pain descriptors of the BPI (left) and total LAI index (right) BPI: Brief Pain Inventory-Short form; LAI: Lequesne Algofunctional Index

Regarding the LAI, most patients were initially classified as "extremely severe," with an average score of 18.17. At the six-month follow-up, there was a slight reduction of 16.84%, averaging a score of 15.11. However, this result may be influenced by one patient showing an 11-point reduction (σ = 31.4%). Patient demographics, BPI-sf, and LAI scores are detailed in Figure 3 and Table 1.

**Table 1:** Analysis of the patients and the outcomes BPI: Brief Pain Inventory-Short form; LAI: Lequesne Algofunctional Index; min: minimum; max: maximum; M: male; F: female; μ: population mean

Case	Age	Sex	BPI max	Six-month follow-up	BPI min	Six-month follow-up	BPI μ	Six-month follow-up	BPI now	Six-month follow-up	LAI total	Six-month follow-up
1	79	M	9	8	9	6	9	5	9	3	19,5	16.5
2	43	F	8	0	4	0	6	0	8	0	11	0
3	63	M	9	9	0	0	7	5	7	2	18	17.5
4	73	M	10	7	3	1	5	5	10	3	24	20
5	82	M	9	2	0	0	6	1	6	0	12	10.5
6	72	M	9	7	5	5	7	5	5	3	20	18
7	73	F	9	6	0	0	7	5	8	4	21	18
8	74	F	10	10	5	1	6	4	6	0	20	19
9	75	M	7	6	2	0	5	1	5	0	18	16,5
μ	70.44	-	8.88	6.11	3.11	1.44	6.44	3.44	7.11	1.67	18.17	15.11

## Discussion

The predominant minimally invasive intervention for GTPS has been local corticosteroid injections. However, while initially showing superiority over a "wait and see" approach in the short to mid-term, its efficacy appears comparable to education plus exercise therapy at the one-year mark [13, 23]. Moreover, emerging evidence suggests the potential long-term benefits of alternative therapies like shockwave therapy and platelet-rich plasma injections [11, 13, 24, 25], although this evidence is based on limited data.

Amidst these considerations, exploring novel therapeutic options became imperative. In 2012, Genth et al. [20] identified a small nerve, termed the trochanteric branch of the femoral nerve, responsible for the sensory innervation of the greater trochanter. Their findings contradicted Dunn et al.'s assertions [26] about a sensory branch from the inferior gluteal nerve, suggesting a possible anatomical variation.

Our study demonstrated favourable outcomes in most patients, with an average reduction of 76.51% in symptoms as measured by the BPI-sf question, "How much pain do you have right now?" Eight out of nine patients experienced at least 50% relief, akin to Abd-Elsayed et al.'s findings [27], which reported an average pain reduction of 71.4%. Notably, our six-month follow-up period surpassed their comparatively shorter 56-day timeframe, although both studies only offer evidence for the potential short-term benefits of this intervention.

No immediate complications or adverse effects were observed after the procedure or reported during subsequent follow-ups, consistent with findings from Abd-Elsayed et al. and Chen et al. [27, 28], where complications were notably rare. Despite this, given the technique's novelty, we chose pulsed radiofrequency over ablative options to minimise the likelihood of complications [29, 30].

## Conclusions

This minimally invasive technique seems to be promising, particularly in addressing the substantial population of non-responders to current conservative treatments. Our findings revealed a reduction in symptoms for the majority of patients, with an average reduction of 76.51% in reported pain levels as measured by the BPI-sf question "How much pain do you have right now?".

This result, in conjunction with the absence of adverse effects, underscores the potential efficacy and safety of ultrasound-guided bipolar pulsed radiofrequency for greater trochanteric pain syndrome.

There remains a need for controlled, larger-scale studies to evaluate the effectiveness and long-term side effects of this type of procedure, as well as compare it to the existing treatment modalities, such as corticosteroid injections alone versus a combination of both.
